# Supplementary material for: Identification of photoperiod-induced specific miRNAs in the adrenal glands of Sunite sheep (Ovis aries)
Source: Front Vet Sci. 2022 Jul 22;9:888207. doi: 10.3389/fvets.2022.888207 (PMC9354845; doi:10.3389/fvets.2022.888207)
Supplement: Supplementary file 1 [file Data_Sheet_1.ZIP › Supplementary file/miRDeep2 file.zip!/miRDeep2 file/Know_Structure/LP42A3/oar-mir-25.pdf]

miRBase precursor : oar-mir-25
Total read count : 18852
oar-miR-25 read count : 18760
remaining reads : 92

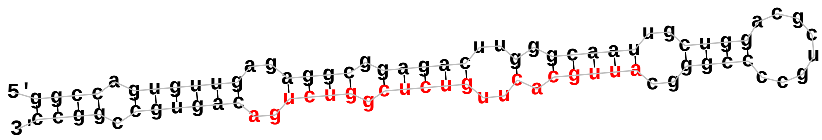

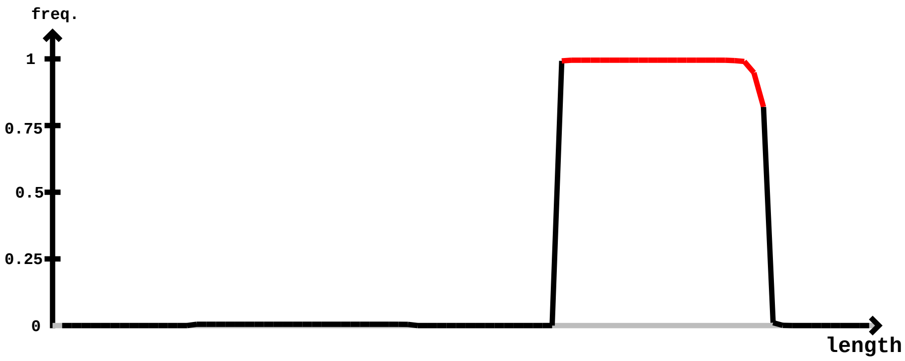

oar-miR-25

5'- ggccaguguugagaggcggagacuugggcaauugcuggacgcugccccgggcauugcacuugucucggucugacagugccggcc -3'	exp		
((((.((((((..((((.(((((..(.(((((..((((........))))..))))).)..))))).)))).))))))).))))	reads	mm	sample
............aggcggagacuugggcaauug...	1	0	seq
............aggcggagacuugggcaauugc..	8	0	seq
............aggcggagacuugggcaauugcu...	83	0	seq
....gcauugcacuugucucggucuga...........	2	0	seq
....cauugcacuugucucgg................	2	0	seq
....cauugcacuugucucggu...............	28	0	seq
....cauugcacuugucucgguc..............	60	0	seq
....cauugcacuugucucggucu.............	790	0	seq
....cauugcacuugucucggucug............	2424	0	seq
....cauugcacuugucucggucuga...........	15203	0	seq
....cauugcacuugucucggucugac..........	186	0	seq
....cauugcacuugucucggucugaca.........	15	0	seq
....auugcacuugucucggucu.............	2	0	seq
....auugcacuugucucggucug............	3	0	seq
....auugcacuugucucggucuga...........	41	0	seq
....auugcacuugucucggucugac..........	1	0	seq
....uugcacuugucucggucuga...........	1	0	seq
....ugcacuugucucggucuga...........	1	0	seq
....gcacuugucucggucuga...........	1	0	seq
